# Supplementary material for: Nanomotion technology for testing azithromycin susceptibility of Salmonella enterica
Source: Microbiol Spectr. 2025 Apr 24;13(6):e02385-24. doi: 10.1128/spectrum.02385-24 (PMC12131866; doi:10.1128/spectrum.02385-24)
Supplement: Supplemental material — Fig. S1 to S8; Table S1. [file spectrum.02385-24-s0001.docx]

### Supplementary materials

**Nanomotion technology for testing azithromycin susceptibility of *Salmonella enterica***

Authors: Mariliis Hinnu^1^#*; Toomas Mets^1^; Ivana Kerkez^1^; Marta Putrinš^1^; Niilo Kaldalu^1^; Gino Cathomen^2^; Marta Pla Verge^2^; Danuta Cichocka^2^; Alexander Sturm^2^; Tanel Tenson^1^; for the ERADIAMR consortium§

^1^Institute of Technology, University of Tartu, Tartu, Estonia

^2^Resistell AG, Muttenz, Switzerland

#Address correspondence to Mariliis Hinnu, [mariliis.hinnu@ut.ee](mailto:mariliis.hinnu@ut.ee)

Running Head: Nanomotion-based AST for azithromycin in S.enterica *[limit: 54 characters and spaces]*

*Present address: Institute of Molecular and Cell Biology, University of Tartu, Tartu, Estonia

§The ERADIAMR (Effective RApid DIagnostics and treatment of AntiMicrobial Resistant bacteria) is a European project on antimicrobial resistance part of the JPI-AMR action. The ERADIAMR consortium is composed of the following persons:

-        Christèle Aubry, Amanda Luraschi-Eggemann, Maria Georgevia & Gilbert Greub, Lausanne, Switzerland

-        Gino Cathomen, Danuta Cichocka & Alexander Sturm, Muttenz, Switzerland

-        Maria Garcia-Castillo & Rafael Canton, Hospital Ramón y Cajal-IRYCIS and CIBER de Enfermedades Infecciosas (CIBERINFEC). Madrid, Spain

-        Nicolas Oswaldo Trinler & Susanne Häussler, Helmholtz Centre for Infection Research, Germany

-        Mariliis Hinnu, Niilo Kaldalu & Tanel Tenson, Institute of Technology, University of Tartu, Estonia

-        Tailise de Souza Guerreiro Rodrigues & Stefano Pagliara, Living Systems Institute, University of Exeter, UK

### Supplementary figures

Figure S1. Influence of incubation temperature and growth rate of wt *Salmonella* on the MIC of AZI. A. Bacteria are more sensitive to AZI at room temperature (23C). Paired t test revealed statistical significance (two-tailed p=0.0073) between 23°C and 37°C across all media. B. The MICs in different media do not correlate with the generation time (G) of bacteria in media without AZI. Generation time was calculated between 1 and 3 hours of static growth at 37°C. LP = low phosphate medium with 980 µM magnesium; LPM = low phosphate & low magnesium (49 µM) medium. MOPSglc = 1XMOPS with 0.2% glucose; MOPSgly = 1XMOPS with 0.2% glycerol; CAA = cas-amino acids. All media were buffered with 40 mM HEPES at pH 7.4. Means ± SD (N ≥ 3 biological replicates) are shown.

Figure S2. Nanomotion variance signal measurements of wt *Salmonella* with 16 or 32 µg/ml AZI for 2 hours and subsequent recovery in fresh medium at room temperature (RT) or at 37°C. Means ± SEM (N≥2).


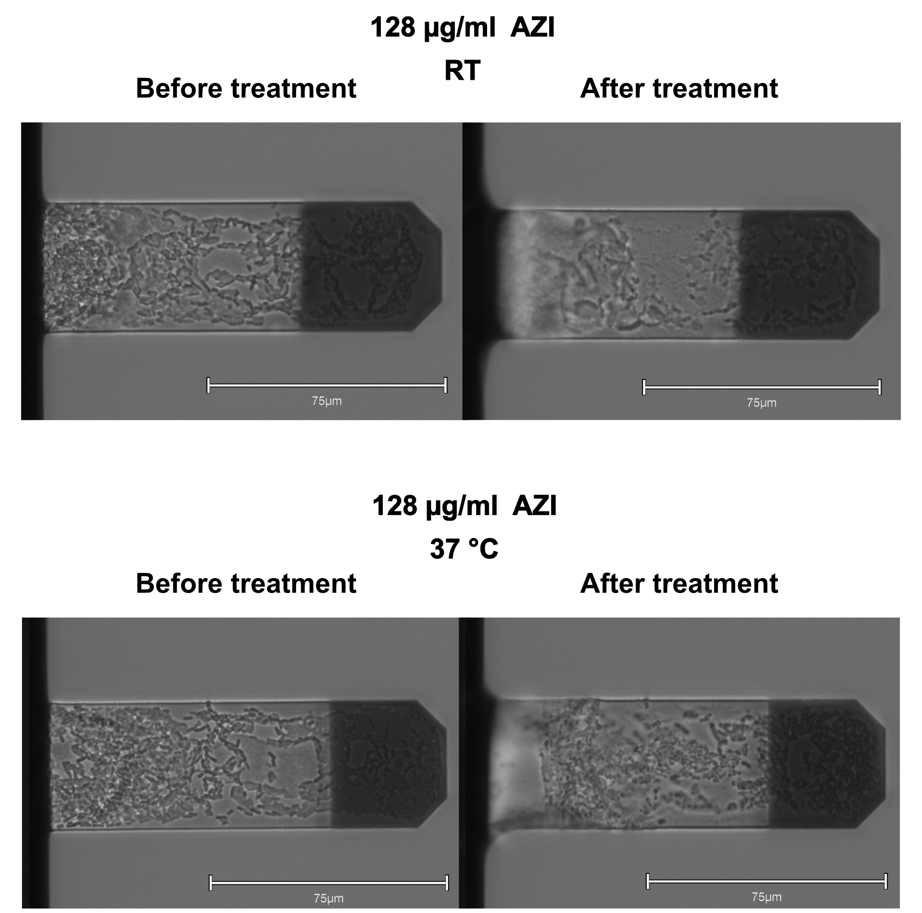


Figure S3. Phase contrast images of cantilevers from recovery experiments before and after 128 µg/ml AZI treatment. Bacterial cells are present on the cantilever after the treatment period at both temperatures. Cells did not recover at RT, but did recover at 37°C. Representative images from single experiments.


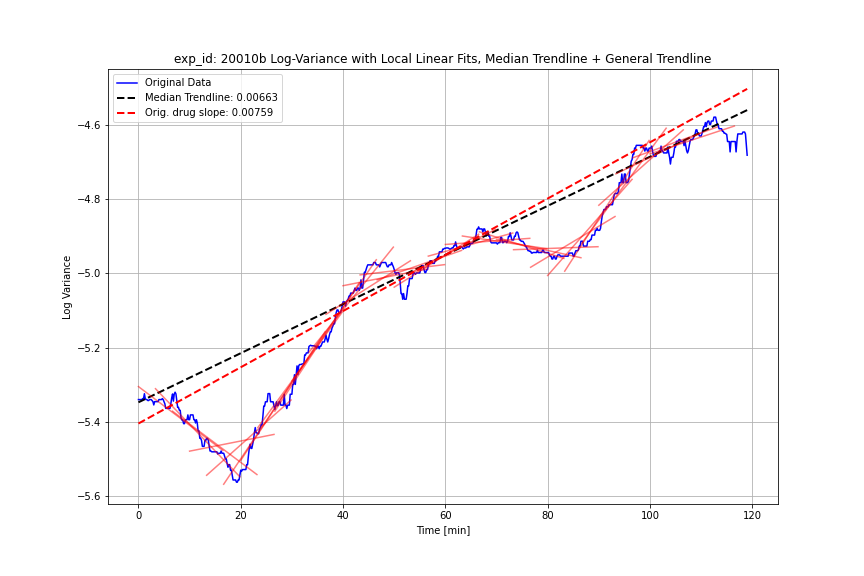

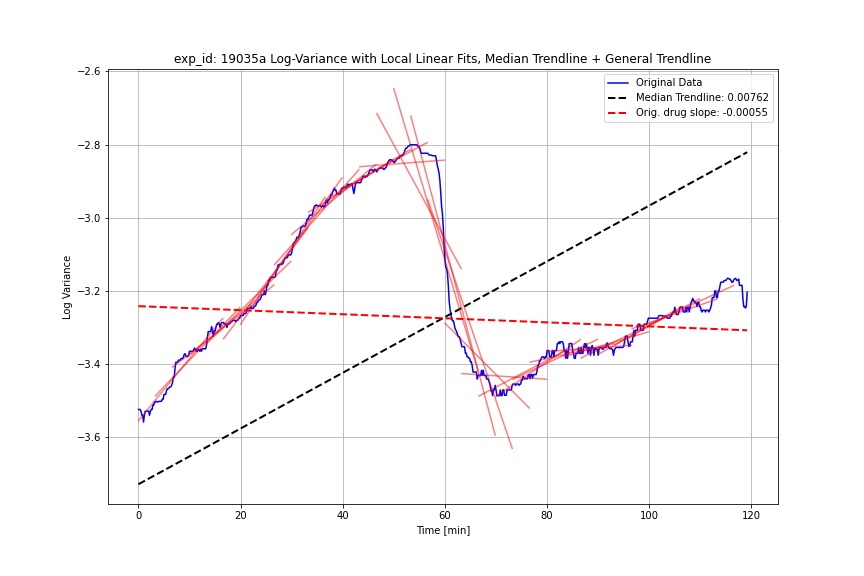


Figure S4. *Robust slope estimation with rolling regression.* Blue line: Log10-Variance curve (unitless). Red-dashed line: Slope over the entire 120-minute drug phase. Black-dashed line: Median of locally fitted slopes. Red straight lines: Local linear fits. In the top panel, it is shown that the difference between the robust estimate (black-dashed line) and the slope over the whole phase (red-dashed line) is only marginal in standard cases. The bottom panel demonstrates cases where the slope over the whole signal fails to deliver a sensible result, whereas the robust estimate (black-dashed line) provides a reliable and meaningful estimate. The y-axis is on a logarithmic scale. These figures support the reliability and robustness of the Rolling Regression method.


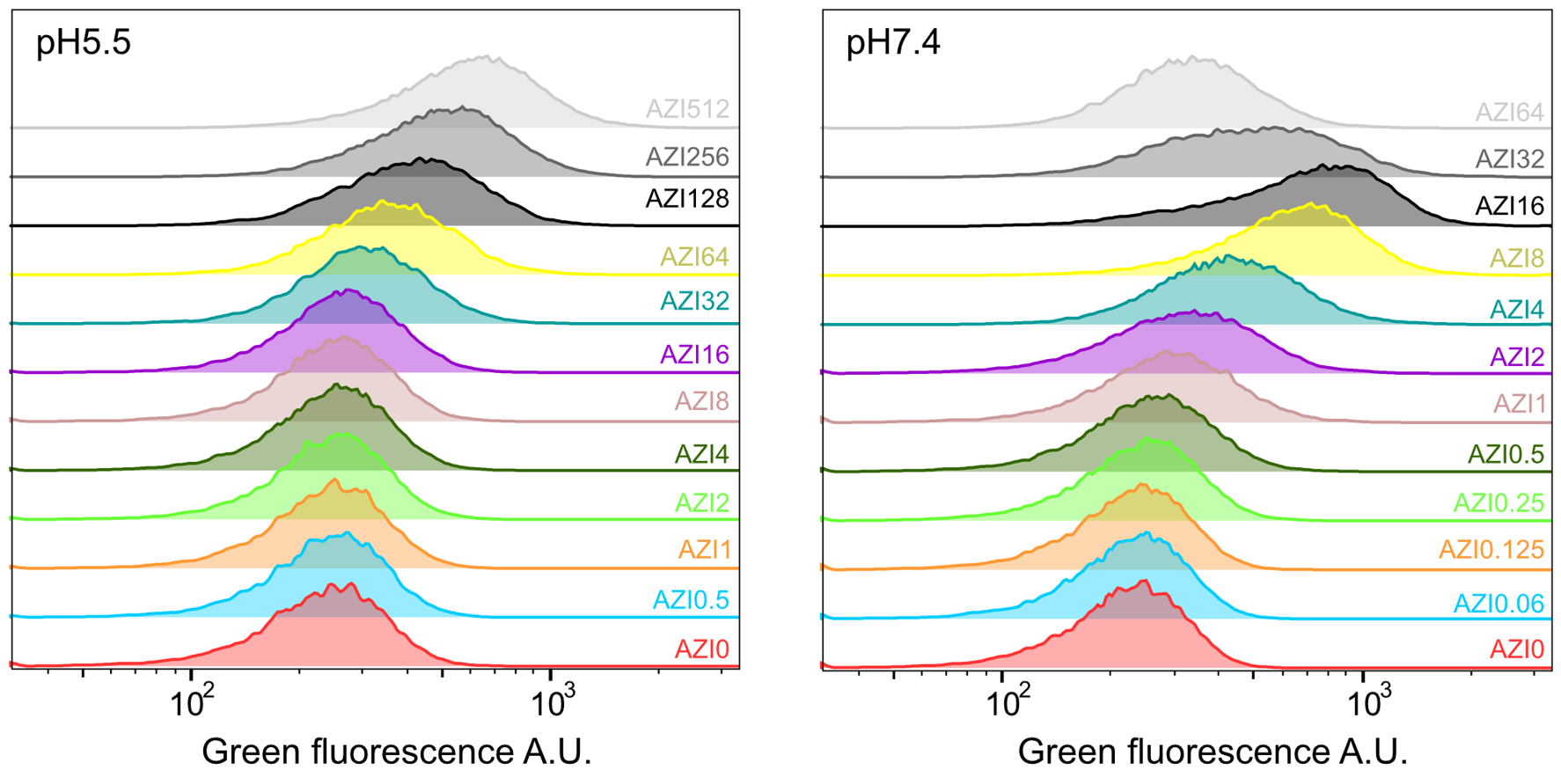


Figure S5. Macrolide-inducible ErmCL-GFP reporter in wt *Salmonella* after 2 h of treatment with AZI at 37 °C. Histograms from flow cytometry data show normal distribution of green fluorescence. Representative histograms from a single experiment. Number behind AZI indicates concentration in µg/ml.

Figure S6. Dose-responsive induction of the ErmCL reporter with AZI. At 37°C the reporter is induced at a concentration about ¼ of MIC. At 25°C the reporter induction is visible at about MIC concentration, however, the induction levels remain much lower probably due to slower metabolism. Numbers in the legend indicate AZI concentration in µg/ml. Platereader data. Means ± SD (N = 4).

| 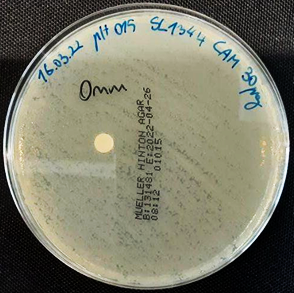 | 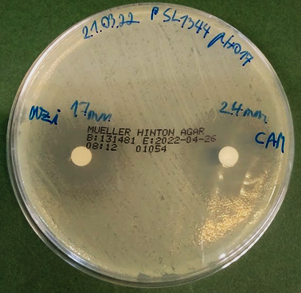 |
| --- | --- |

Figure S7. Antibiotic activity assessment based on disc diffusion assay on wt *S. enterica* SL1344. Left image: lack of antibiotic activity of an inactive form of chloramphenicol (CAM, 30 µg per disc) as no inhibition zone is visible on an agar plate. Right image: inhibition zones of AZI (left, 15 µg per disc) and active CAM (right, 30 µg per disc). Resistance breakpoints for Enterobacteria are <12 mm for AZI and <17 mm for CAM (11).


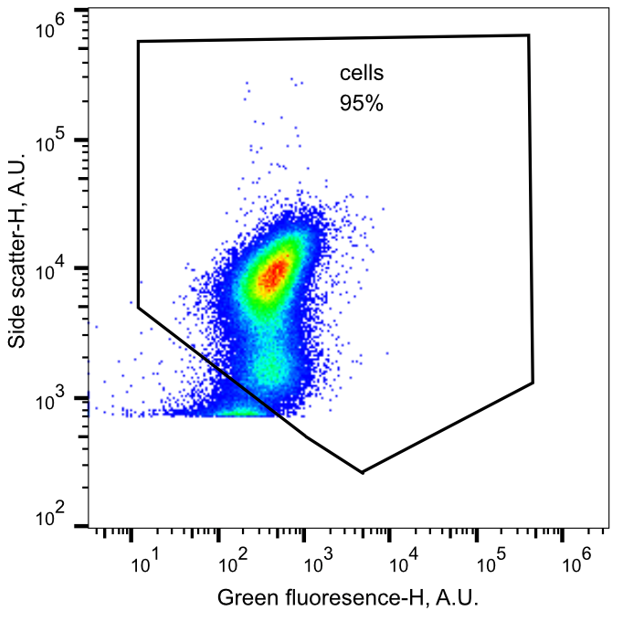


Figure S8. Gating strategy of flow cytometry data. Bacterial cells were separated from flow cytometry noise according to forward scatter and green fluorescence. Representative dotplot from single sample (pH7.4, AZI 4 µg/ml). Gates were manually adjusted for each sample to exclude the noise, mainly taking into account the side scatter.

Table S1. MIC-s of azithromycin in different conditions.

| **Salmonella strain** | **Medium** | **Buffer** | **MIC* (µg/ml)** | |
| --- | --- | --- | --- | --- |
|  |  |  | **23°C** | **37°C** |
| SL1344 (wt) | MHB | 40 mM HEPES pH7.4 | 2 | 4...8 |
|  |  | 100 mM HEPES pH7.4 | 2…4 | 8 |
|  |  | 100 mM MES pH 5.5 | 512...1024 | >1024 |
|  | LP | 40 mM HEPES pH7.4 | 0.25 | 1 |
|  | LPM | 40 mM HEPES pH7.4 | 1 | 2 |
|  | MOPSglc | 40 mM HEPES pH7.4 | 2 | 4 |
|  | MOPSglcCAA | 40 mM HEPES pH7.4 | 2 | 4 |
|  | MOPSgly | 40 mM HEPES pH7.4 | 1...2 | 4 |
|  | MOPSglyCAA | 40 mM HEPES pH7.4 | 2 | 4 |
| SL1344 *acrB* R717Q | MHB | 100 mM HEPES pH7.4 | 4...8 | 32 |

*MIC-s from at least 3 biological replicates given as a single concentration, if replicates were equal, or as a range.

LP = low phosphate medium with 980 µM magnesium; LPM = low phosphate & low magnesium (49 µM) medium; MOPSglc = 1XMOPS with 0.2% glucose; MOPSgly = 1XMOPS with 0.2% glycerol; CAA = cas-amino acids
